# Promoting the mental health and well-being of vulnerable youth through art: an ethnographic evaluation of an art-based intervention for rural Canadian youth

**DOI:** 10.1186/s40359-025-02476-8

**Published:** 2025-03-03

**Authors:** Kyra Simons, Adrianna Mendrek, Jasmine Piché, Megan Bernier, Terra Léger-Goodes, Catherine Malboeuf-Hurtubise

**Affiliations:** 1 Bishop’s University (Psychology), Sherbrooke, QC Canada; 2 University of Quebec at Montreal (Psychology), Montreal, QC Canada

**Keywords:** Youth, Art-based interventions, Mental health, Well-being, Community-based research

## Abstract

**Background:**

Children in rural communities are of the most vulnerable populations in Canada. Living in a rural community has mental health and well-being implications for these children, particularly regarding their access to mental health services. By comparison to talk therapies, which are often costly, art-based interventions are accessible financially, geographically (i.e., offered in academic settings), and across abilities. Qualitative research of art-based approaches with vulnerable children is limited. Despite this, art-based interventions have allowed children to express their feelings through art, which in turn helped them identify and verbalize thoughts and feelings; something that youth tend to struggle with in traditional psychotherapy. As such, considering the vulnerable context of the students in the present research, a community-based approach was deemed beneficial—and thus adopted—in the current project as we created art with underprivileged children in a rural community with a specific focus on promoting positive mental health.

**Aim:**

The purpose of this project was to examine the experiences and preliminary impact of an art-based intervention with students in rural communities.

**Methods:**

27 youth from Quebec, Canada, aged 10–12 took part in a weekly art-based intervention for 7 weeks that was designed to cater to their unique needs. The intervention design and specific art activities were chosen according to the community partners’ description of the students’ needs and previous work led by the research team which aimed to improve elementary school children’s mental health. An ethnographic approach was used to examine the implications this intervention had on students, particularly their mental health and well-being. Thematic analysis was used to examine the data.

**Results:**

The majority of students reported enjoying the intervention, many stating it had a positive impact on their lives. For example, various students reported that the intervention made them feel more comfortable amongst their peers and program staff. Students also reported feeling positively during art-making and expressed interest in taking part in follow-up research.

**Discussion:**

Overall, positive experiences with the present intervention support existing literature regarding the effectiveness of art-based methods for youth populations. Future research should further investigate the importance of the therapeutic alliance in youth community- and art-based research, particularly how the age of the facilitator can enhance rapport. Additionally, further research is needed to understand how art-based interventions, although sometimes unenjoyable, can have positive implications through pushing personal limits.

**Supplementary Information:**

The online version contains supplementary material available at 10.1186/s40359-025-02476-8.

## Introduction

### Access to mental health services

Globally, youth experiencing mental health concerns have consistently been underserved, with less than half of these youth receiving the mental health services they need [1, 2]. Moreover, only 20% of the 1.2 million Canadian youth experiencing mental health issues are receiving appropriate care and treatment [3].

One of the most notable difficulties regarding youth populations receiving mental health services is a lack of youth-friendly programming [2]. It thus seems imperative to invest in early interventions for young people that aim to prevent mental illness and promote mental health [2, 4]. In fact, 70% of mental health problems emerge in childhood and adolescence [5].

Mental health prevention programs should be effective, well-adapted, and accessible to ensure implementation and effectiveness. Commonly accepted approaches to therapy with children are vast and well-reviewed, yet lack availability [6]. For example, cognitive behavioural therapy has proven to be effective amongst a wide variety of youth populations [7, 8], but it is not as easily accessible for low-income individuals [9]. Indeed, youth of low income families experience social-emotional issues linked to chronic stress which are exacerbated by a lack of access to care [10]. Conversely, unconventional approaches to treatment such as art-based interventions have been less studied, but have the potential for wider availability [6]. These interventions are an integrative mental health service that engages participants through art-making, creative process, applied psychological theory, and human experience [11]. Further, they have the capacity to support both personal and relational treatment goals, as well as community needs [11]. As mentioned, art-based interventions are more accessible than traditional therapeutic approaches [9]. For example, the wider availability of art-based interventions allows individuals to experience the positive effects of art making at home without the presence of the therapist [12]. However, there are varying perspectives regarding the effectiveness of art-based approaches [13, 14]. For example, the qualifications of art therapists and the effectiveness of art therapy has been underappreciated by psychiatrists (see [13], while other research has found that psychotherapists view art therapy as a legitimate form of psychotherapy (e.g., [14].

### Effectiveness of art-based interventions

Although research regarding the effectiveness of art-based interventions alone amongst youth populations is emerging, it has shown to improve quality of mental health and symptoms of posttraumatic stress disorder amongst adolescents [15]. Within this work, 470 students in Nigeria were divided into control, fine art therapy, and music therapy groups, and it was found that fine art therapy was more effective than music therapy at reducing PTSD symptoms [15]. In another study, youth aged 10–12 of low income who participated in a 12-week drumming intervention exhibited significant improvements in their overall social-emotional behaviour [10]. Thus, these positive findings support the effectiveness of art-based interventions, and this is also true when it is complemented by conventional approaches such as CBT and DBT [16].

By comparison to conventional therapies, there are various unique attributes to art-based interventions [11]. Namely, art-based interventions acknowledge our subjective experiences through a creative process [11]. Being creative can have a positive impact on mental health and well-being [17]. For example, being creative enhances meaning-making [18] and satisfaction in daily life amongst individuals experiencing severe mental illness [19]. Conversely, well-being has also been found to be conducive to creativity; highlighting the reciprocal relationship between the two constructs [17]. Positive emotions, such as those experienced when being creative [17], expand individuals’ thoughts and actions; motivating them to think differently without fear. Positive emotions promote creativity, which in turn builds one’s psychological, physical, social, and intellectual resources [20].

Engaging in the creative process through art-based interventions have served to overcome youth populations’ resistance to therapy [21] and relieve the delays associated with seeking conventional therapy [6]. Art-based interventions can be particularly beneficial for children who may not possess the vocabulary to thoroughly describe their emotions [20]. Indeed, art therapies can facilitate unique, nonverbal communication for youth who experience trouble verbally communicating their thoughts and feelings [20]. Although there is a lack of research comparing the effectiveness of art-based interventions to more conventional forms of therapy, art-based therapies have been reported to be the preferred mode of therapy by youth who have experienced mental health concerns [22], making it a suitable and promising intervention for vulnerable populations.

### Art-based intervention amongst vulnerable populations

Art-based interventions are particularly beneficial for vulnerable populations, namely youth, immigrants, refugees, and individuals of colour [23–27]. Specifically, art-based interventions are beneficial for these populations as it circumvents language barriers and focuses on positivity and growth [28]. Moreover, art-based interventions allow individuals to explore memories and emotions, which provides a safe space to overcome the effects of trauma [28]. Among youth, immigrants, refugees, and individuals of colour, art-based interventions have proven to decrease symptoms of depression and stress [24, 25, 27], and enhance positive emotions such as hope and resilience [23, 24]. Research regarding interdisciplinary group programming for adolescents in inpatient care found that participants preferred taking part in creative activities and art-based interventions to verbal psychotherapy, which they found to be too confronting [22]. Despite this preference for engaging in the arts, there is little to no research regarding which art-based methods are most effective amongst youth.

To navigate this gap in the literature, participants can often inform the art-based interventions themselves [24]. Through collaborative research designs, investigators can tailor the intervention so that it is culturally and age appropriate, and aligns with the context of participants [24, 26]. The vast mediums within art allow for psychologists to adapt their interventions according to the demographic they are working with, which is particularly important within art-based interventions [26]. With regards to working with youth populations specifically, art-based interventions allow psychologists to engage children while giving them a sense of control through the creative process [29].

According to Boaz and Bat-Or (2022), involving participants in the creation of research, known as taking a community-based approach, attempts to create a safe space for communal art-making while integrating participants’ needs and wishes. Art-making in a group setting can also bring a community closer through sharing similar experiences with one another during sessions [26, 30]. A community-based approach was deemed particularly beneficial—and thus adopted—in the current project as we created art with underprivileged children in a rural community with a specific focus on promoting positive mental health.

### Study aim

The overarching goal of this project was to develop an art-based intervention for vulnerable youth attending an after-school program. Specifically, through our qualitative design, we aimed to document students’ responses and perceived benefits of engaging in artistic creation on their overall mental health. The guiding research questions were “which artistic mediums and psychological themes will students be positively responsive to?” and “how will vulnerable youth benefitting from an art-school program respond to an art-based intervention aimed at improving their mental health and well-being?”.

## Materials and methods

The present study obtained approval from the Bishop’s University Ethics Board on March 17th, 2023 (102,681), which adheres to the 2018 Tri-Council Policy Statement: Ethical Conduct for Research Involving Humans (TCPS 2) and the Bishop’s University Research Ethics Policy.

## Research team and reflexivity

This research was led by the first author (KS, a PhD student in psychology). KS received training in qualitative methods through completing masters-level courses in qualitative psychology and from her qualitatively-trained colleagues. Prior to this research, KS had played an active role in various research projects under the supervision of CMH, including research on the impact of studying positive psychology, and the psychological implications of climate change anxiety on children. This project was KS’ first project as lead investigator. She conducted this work with the help of two undergraduate research assistants, who had each received provincial funded scholarships for undergraduate initiation to research. This research team was supervised by CMH and AM, who have made considerable contributions to the fields of child and adolescent clinical psychology, neuroscience and mental health respectively, amongst other topics including art-based research.

KS met with the program coordinators various times prior to the onset of the project to develop her understanding of the research participants, the participants’ needs, and to collaboratively develop an intervention plan alongside the program coordinators. However, the research team did not meet with any of the potential participants when assessing these factors.

The research team’s intention with the project was to develop an intervention that adequately addressed participants’ needs so that participants – who, according to the program coordinator, faced various behavioural, emotional, and development challenges and lacked access to psychological services – could experience positive psychological implications.

### Participants

The current project was conducted in an English-speaking town in rural Quebec, which is ranked among the lowest communities on Quebec’s Socioeconomic Environment Index. Participants attended schools that were amongst the most disadvantaged in the province where the majority of families in this area have been considered below the low income threshold index [31].

Youth taking part in this study faced four prominent challenges: deprivation and vulnerability, language challenges, a lack of role models, and isolation and stigma. Many of the students who attended the after-school program—and took part in this study—experienced emotional, behavioural, and/or developmental challenges, making them more likely to have significant mental health care needs [32]. Noteworthy, many of the study participants were not bilingual (and have English as their mother tongue). However, in the province of Quebec, where French is the official language, access to appropriate mental health interventions is particularly challenging for youth who belong to a linguistic and cultural minority [9].

### Intervention

The art-based intervention used in this work was developed by a child psychologist (CMH) and a M.Sc. student in psychology (KS), in collaboration with a researcher who has extensive training in dance and movement therapy (AM). The theoretical approach to this work, and to the selection of specific activities was directed by both pragmatist epistemology and participatory action research theory. A pragmatic paradigm is associated with a plurality of methods, asserting that researchers should implement the philosophical and/or methodological approach that is most effective for the particular research problem or group being investigated [33]. In a similar vein, pragmatist epistemology asserts that our knowledge and perception of the world are based on experience [33]. Pragmatist epistemology is similar to participatory action research as they maintain that without action, we do not know what is useful or desirable for participants [34, 35]. Participatory action research is conducted with the intention of transforming the lives of socially marginalized populations [33].

Our intervention was guided by both pragmatist epistemology and participatory action theories. Indeed, weekly themes and activities were chosen based on what was deemed impactful in previous work led by the research team, which aimed to improve elementary school children’s mental health [36]. The current intervention was reviewed by the partnering program’s coordinators and was adjusted with their feedback in mind. The 7-week intervention included various themes (e.g., relationships, emotions, optimism) which were chosen based on our understanding of the students’ needs. A variety of art mediums (e.g., drawing, dancing, sculpting) were chosen so that participating students could express themselves creatively through different means. See Table 1 for a complete overview of the intervention. Workshops were facilitated by a master’s student (KS) and undergraduate psychology students, all of whom were supervised by a child psychologist (CMH). Each twenty-five minute session focused around a single theme and proceeded as follows: instructions, artistic creation, group discussion and sharing of created art. The art-based activities were selected to encourage introspection and creative expression in students.

**Table 1:** Breakdown of art-based activities

Intervention week	Art medium	Theme	Activity
1	Drawing	Emotions	“On a pie chart, illustrate and colour the following emotions.”
2	Collaging	Identity	“Using magazine clippings, create a self portrait.”
3	Dance	Coping	“Choose three movements that represent overcoming a challenge. Share those movements with a peer.”
4	Sculpture	Safe space	“Using Lego, build a space where you feel safe and calm.”
5	Writing and drawing	Relationships	“Write a poem or letter about a loved one in your life, your pet, or your favourite food. If time allows, illustrate your letter.”
6	Meditation and drawing	Hope and optimism	“Relax, and get comfortable in your spot. Listen to the following meditation. Afterwards, illustrate the scene you visualized.”
7	Drawing	Summary of project	“While taking turns completing the individual interviews, please illustrate an image that represents the time we spent together and the activities we completed.”

### Study eligibility

Study participants included three groups of 5th and 6th grade elementary school students (*n* = 27; aged 11 and 12 years old), divided between English speakers (*n* = 9) and French speakers (*n* = 18), all of whom attended an after-school program located at a nonprofit center. Factors for vulnerability among these students included, but were not limited to: low socio-economic status, low level of education among parents, single-parent families, high incidence of mother abandonment, high level of learning and behaviour difficulties, and lack of access to essential services in English (speech therapy, social workers, etc.). According to the program coordinator, some of the students had experiences with psychologists and social workers. In her opinion, although she could not confirm, many of the other students had as well. We did not include this in the manuscript for one key reason: the program coordinator was not included as a participant in the project, and therefore we did not consider her comments as research data. Additionally, we did not screen for participants’ diagnosed/probable mental health conditions as they were too young for us to administer a quantitative assessment at pretest [37], Sect. 5).

Recruitment was non probabilistic, through our research partnership with the program. Specifically, the program coordinator presented the study to parents of children attending the program and obtained informed consent for their child to take part in the intervention and an individual interview at the end of the study.

### Design

An ethnographic evaluation was conducted in this preliminary study. Data was obtained through semi-structured interviews with the students, as well as through group discussions and observational data of the workshops. Through qualitative research, one can further investigate how to optimally facilitate and test proposed interventions [38]. The efficacy of interventions that are not yet empirically validated can be explored through qualitative feedback, as we collect data on what worked and didn’t work for participants.

Through working alongside the coordinators of our partnering program throughout the research process, we were able to curate the intervention design so that it best served our population of interest. Specifically, certain themes were chosen with the intention of uplifting participants and helping them realize their full potential, and the intervention plan was approved by the program coordinators prior to the onset of data collection. Further, the weekly activities were altered on occasion if students were not initially receptive to them.

### Data collection

Data was obtained from various sources: researcher observations, group discussions following each workshop, and individual semi-structured interviews with students at the end of the intervention. The 2 undergraduate research assistants who assisted in data collection were trained by KS, CMH, and AM.

Observations were collected throughout the intervention by the assisting undergraduate researchers. They focused on topics such as students’ reactions to the weekly activity, non-verbal messages from students, and the interactions that occurred between students during the activity. See Table 2 in Appendix A for the observation grid our team used.

Following each activity, as a group, all students were asked about what they had created, if they wished to share their creation, their reactions to the activity, and how the activity may have related to their everyday lives. Group conversations were conducted weekly with the intention of receiving participant feedback in a timely manner, rather than weeks later in the individual interviews. Group discussions were recorded and transcribed.

After seven weeks of group activities, each student took part in a brief, semi-structured interview at the center, which was led by a member of the research team. Similarly to the group discussions, these conversations were also recorded and transcribed. During the interview, students were asked questions regarding their perception of the intervention, their appreciation of specific activities, and the degree to which they felt the intervention had an impact on themselves or their lives. See Appendix B for the complete interview guide, which was created by KS, CMH, and AM, and was based on previous research that was conducted within our lab. Students were not compensated for their participation as our research team would help them with their homework in the half hour that followed each weekly activity. All interviews were recorded and transcribed for analysis. As all activities and interviews with the French students were conducted in French, these data were translated to English prior to analysis.

### Analysis

Collaborative thematic analysis was conducted between researchers (KS and JP) to analyze the individual interviews. Thematic analysis [39] was used to allow for participants’ perceptions and experiences of our intervention to emerge, enabling our research team to grasp the impact the intervention had on participants. The transcriptions were analyzed using NVivo 14. Transcripts were read repeatedly by two researchers (KS and JP) prior to the initial open coding phase. Codes were determined inductively and defined early on in analysis. These codes were discussed with and reviewed by two other research team members (CMH and AM). The two researchers coded the data independently but frequently discussed observations, allowing them to collaboratively structure codes and deduce arisen themes. Themes were then defined.

Following the thematic analysis of the individual interviews, group interviews were analyzed using both inductive and deductive content analysis. An inductive approach was used to interpret themes that emerged from observational data. Then, deductive analysis was conducted based on the codes and observations that emerged from the thematic analysis of the individual interviews. Compared to the first round of inductive thematic analysis, this second round of inductive thematic analysis allowed both code elements from the initial coding tree (deductive), and report coding frequency to provide an idea of the number of times certain elements were mentioned in the data. This type of qualitative analysis allowed researchers to analyze both the existence of themes within group conversations, and the frequency in which these themes occurred in the data. By using both inductive and deductive approaches, researchers could apply what emerged inductively from the observation data to the group conversation so that we could enrich our understanding of resulting concepts from both the individual and group discussions.

## Results

See Table 3 in Appendix A for the summarized themes, student verbatims, and examples from data collection.

### Engagement in the intervention

Overall, students’ engagement in the intervention primarily depended on their general enjoyment of art and their commitment to the intervention. Specifically, those who described disliking creating art and/or feeling the intervention was pointless appeared much less likely to be actively engaged in the intervention than those who described enjoying art-making and/or feeling as though the intervention was important.

The majority of students felt positively about the intervention, and indicated that they enjoyed taking part in the weekly activities. Students typically reported having fun throughout the intervention. Participants were perceived to enjoy taking part in the intervention, as evidenced by students often complaining when our allotted activity time was over and they had to move onto doing homework. Some students were reluctant to participate in the intervention at times, explaining that they had a lot of homework to do. However, these students were enthusiastic about participating on weeks they did not have homework to complete.

Additionally, observation revealed students’ energetic behaviour and lack of time as other challenges that inhibited participation in art-making. Specifically, students often arrived at the after-school program with copious energy and were often unwilling to listen to our research team or the program coordinators. Some children settled within the early minutes of an activity, however many others continued to be disruptive and were reluctant to participate, posing negative implications for those in the room who were focused on the given activity. Moreover, the time we were allotted to lead each art activity and follow-up discussion as a group proved to have limitations on students’ participation.

Other challenges that students described negatively impacting their engagement were lacking inspiration on what to create and feeling as though the intervention pushed them out of their comfort zone. Indeed, a few students described feeling embarrassed when asked to share their artwork. By contrast to this feedback and the researchers’ observations, most students did not feel that there was anything challenging their ability to take part in the intervention.

Furthermore, conversations amongst the French groups led to discussions regarding art-making. When asked how they felt during and after art-making, the vast majority of students mentioned feeling positively. More specifically, of the eight students who were asked, seven felt positively both during and after art-making. Specific emotions discussed amongst those who felt positively during and/or after art-making include happiness and peacefulness. By contrast to those who felt positively during art-making, six students mentioned feeling embarrassed or bored at at least one point of art-making. Students who felt negatively typically attributed their feedback to feelings of embarrassment about the art they created, often stating that they felt they lacked artistic skill. Of those who expressed feeling bored, however, one student shared that they thought the intervention was pointless because they had done all of the art mediums included in the intervention before.

Throughout individual interviews that followed the intervention, a few students, most of whom were in the English group, appeared to either share mixed feelings or lack introspection about the intervention. Specifically, when asked about their overall perception of the intervention, these students reported experiencing positive emotions as a result of participating in the intervention, but later stated that the intervention had no impact on themselves and their emotions. Some of the students who shared that they did not enjoy the intervention later mentioned that they would participate in the study again. Overall, we feel that the students’ mixed feelings and/or lack of introspection about the intervention were indicative of the variation in the students’ engagement.

#### Students’ engagement with specific activities

By contrast to their overall engagement in the intervention, this section pertains to students’ attitudes surrounding specific activities and their perceptions of the art that was created. As per Table 1, many artistic mediums were included in this intervention. Students had a variety of different favourite and least favourite activities, and often attributed their choice to the medium of the activity (e.g. dance), rather than the topic the activity explored (e.g. coping). Dance and lego were most frequently referred to as students’ favourite activities. However, dance was also most frequently mentioned as students’ least favourite activity. In addition to dance as a common least favourite activity, students also referred to the writing activity, stating that they felt embarrassed writing a personal letter. The meditation was also frequently cited as students’ least favourite activity because other students were disruptive during the guiding of the meditation. Overall, however, a considerable number of students said that they liked all of the activities the same.

Generally speaking, the students who were enthusiastic about participating in the activities chose to distance themselves from those who were being disruptive. However, students appeared far more willing to participate in an activity if they enjoyed its medium. For example, all students participated in the safe space activity where students built a lego art piece. Students in the French group were observed to be generally more willing to participate in the art activities and share their thoughts during group discussions than those from the English group, as evidenced by weekly observation. Furthermore, content analysis of group discussions revealed that the French students appeared to grasp the intent of each activity with greater ease than the English students. For example, without announcing the theme prior to the meditation activity, many of the French students created within the theme and expressed emotions related to this undisclosed theme, while the English group required greater explanation and guidance from the researchers to grasp the theme.

Additionally, although activities were guided, students had varying interpretations of directions and what to create. An example of the vast perceptions of the activity topics is the meditation, which followed with a drawing. In this activity, students listened to a script about a sailor lost at sea, who was later able to steer themselves to safety with the guidance of a lighthouse. Without sharing the theme of this activity (hope and optimism), students were tasked with drawing an image they visualized during the meditation. It was our intention to see if students focused more on positive emotions such as hope, or negative emotions such as fear. Despite our direction, some students focused on video game characters, places, or events that they associated with the feeling(s) they felt during the meditation. The varying interpretations and images that arose from the mediation activity demonstrates how, despite given direction in each activity, students felt they could create autonomously.

When describing how they felt about their art, some students felt most proud about the activities where they had the opportunity to outline their favourite things, activities, or places. By comparison, other students felt most proud about the piece of art they felt was most impressive or beautiful. Although most students had an activity they felt particularly proud of, many other students did not feel that any activities were exceptionally important to them. Interestingly, one student said that the activity they were most proud of, the writing activity about relationships, was also their least favourite activity to take part in. Within their letter, this participant expressed their appreciation for their favourite video game character, and shared the strong impact the character has had on their life. Regardless of whether these students mentioned an activity they were particularly proud of, four students were critical of themselves and their artistic ability, referring to their creations as “ugly” or “bad”.

In addition, analysis of discussion regarding students’ created art revealed that participants were particularly expressive when sharing their emotion wheels by comparison to other activities. Specifically, many students described the colours and images they associated with emotions such as fear, anger, and happiness.

Students were observed to be hesitant to participate in the writing and dancing activities, as evidenced by observational data. Indeed, observational data supports the tone of embarrassment from English participants throughout art-making, as many of our notes indicate students repeatedly needing support and encouragement from the staff and research team. By contrast, the French students were generally more willing to participate in the dancing activities. For example, one of the French students mentioned that they learned not to be shy. Additionally, many students were repeatedly hyperactive and did not take activities seriously, resulting in the groups frequently needing to be disciplined by our research team and program staff. Despite the frequently chaotic environment during art-making, two individuals who regularly shared their art with the group during weekly discussions reported feeling heard and respected by their peers when sharing.

In terms of the French participants, observational data suggested that 5th grade students appeared to be more open to creativity than the 6th grade students.

Across both English and French groups, and regardless of whether the student was particularly enthusiastic to participate in the activities, students tended to disregard the weekly theme and instructions provided when art-making.

### Impact of the intervention

This theme describes students’ perceived impact of the intervention. Students generally indicated positive change in their life and skillset. For example, three students referred to becoming more confident and comfortable around others through taking part in the project. In terms of students’ perceived impact of the intervention on their mental health and well-being, students referred to experiencing improvements eight times, while feeling no significant changes in well-being was coded ten times. Although many students did not elaborate on what specific changes they endured, those who did reported feeling more calm or happy because they learned about themselves through taking part in the intervention. Generally speaking, those who tested their personal comfort level by fully committing to the intervention appeared to exhibit more positive implications from the intervention than those who did not put their full effort in or withdrew from certain activities.

Additional feedback included two students expressing gratitude towards our research team for our time and effort in leading these sessions, one of whom stated they were happy that we came and were not “adults”. As such, throughout the intervention many students bonded with our research team, and the coordinators of the program expressed how much the students appreciated and looked up to us as mentors.

## Discussion

This study aimed to examine the experiences and preliminary impact of an art-based intervention with students residing in a rural community in Quebec, Canada. An ethnographic evaluation was conducted, and data consisted of group discussions, observational data, and individual semi-structured interviews with the students. These were analyzed using both inductive thematic analysis and content analysis.

### Engagement in the intervention

Although most students were engaged throughout the intervention, some students showed occasional reluctance to participate in the intervention. Specifically, the embarrassment that some students expressed regarding participating in the intervention can likely be explained by the group dynamic between the students. As evidenced through observational data, students tended to make fun of one another and their work, making those who took the intervention seriously uncomfortable to describe their art with full transparency. This finding was further supported through feedback received in individual interviews, where many students were much more vulnerable than they were with the group. Considering that bullying and victimization have negative implications on the mental health and well-being of youth [40, 41], group dynamics appear to be particularly influential on students' engagement in group interventions. In other words, as students generally ridiculed one another and their creations, it is our understanding that the negative, judgmental environment made students less willing to engage in group art-making. Notably, however, art-based interventions targeted at fostering social-emotional learning have been found to increase empathy, compassion, and emotional awareness in elementary school students [42]. As such, future art-based interventions should aim to create a positive, supportive environment amongst students by implementing themes of social-emotional learning (namely compassion and empathy) into the intervention.

Generally, as noted, those who described enjoying art in the individual interview reported that the intervention was useful to them. Moreover, these students were willing to push themselves beyond their personal boundaries through their artwork and were more willing to participate, as evidenced by researcher observations and individual interviews with students. Additionally, these students were observed as being much more likely to share their art during discussion periods than their peers. This suggests that, within the context of group art-based interventions, comfort level and enjoyment of art influence feelings associated with art-making. Indeed, research amongst 5th graders regarding openness to creativity, which is implied through enjoyment of the arts, supports this notion [43].

#### Students’ engagement with specific activities

Students’ engagement with the activities was often attributed to the medium of the art activity, rather than its theme. Including a variety of art mediums is important so that students can feel challenged by the activity and remain engaged throughout the intervention [36]. When asked about activities that appeared to be particularly challenging, such as the dance activity on coping, students’ enjoyment varied. Many participants enjoyed the activity, while others felt quite embarrassed or were entirely unwilling to participate. In terms of the importance of providing challenging interventions, the dance activity may have been too challenging for some students. Within this activity, students were asked to think of a time where they coped with something challenging, represent overcoming the challenge through three movements, and then share those movements with a partner. As these students experienced emotional and behavioural challenges, and were thus prone to high emotional reactivity and negative affect, it is likely that students exhibited emotional avoidance [44]. However, as evidenced by existing research on emotional avoidance in youth (see [44], it is possible that if students were asked about more positive emotions or experiences during the activity (as opposed to coping), they would have been more willing to participate. Moreover, if students were granted more time to become comfortable with movement or were educated on how it has been used for emotional expression and coping with trauma, and hence felt more connected to movement, they may have been more willing to participate. Indeed, an eight month dance intervention amongst adolescent girls found that dance in a non-judgmental environment resulted in participants feeling empowered, and gave rise to emotional expression, self acceptance, and trust in oneself [45]. Overall, our results indicate that students were more willing to participate and enjoyed the activities where they were not asked to do anything they perceived as too personal or embarrassing.

### Impact of the intervention

Students’ generally positive feelings towards the intervention lend support to the existing argument for the use of art-based interventions with youth as an acceptable means for intervention (e.g., [27, 46, 47]. For example, student feedback which described that the intervention positively impacted their confidence (e.g., pushing them out of their comfort zone by exploring difficult themes) supports the effectiveness of the intervention.

Additionally, students’ feedback that the intervention made them feel more confident, comfortable, calm, and happy mirrors results of previous art-based work amongst youth (e.g., [30, 31], although the interventions used in these studies lasted for an hour. This is much longer than the twenty-five minute sessions implemented in this project [22, 23]. For example, Collins et al. (2023) conducted weekly art therapy sessions for twelve weeks amongst youth in a secure care facility [23]. Participating youth experienced enhanced hope and resilience by 29% and 16% respectively, and it was found that the intervention improved youths’ goals, levels of self-determination, and future orientations [23]. Furthermore, Versitano et al.’s (2023) work, which assessed the effectiveness of an art-based intervention and other group therapies amongst adolescents in inpatient mental health care, was conducted over a four month period [22]. Those in the art-based intervention group reported that their sessions were both enjoyable and helpful, and it was found that the use of creative elements promoted consistent group engagement [22].

In addition to referencing supporting well-being, experiencing self-discovery was discussed by participants who enjoyed the intervention simply due to liking art-making. Indeed, previous work suggests that art-based interventions can be conducive to self-discovery in youth [48]. Specifically, adolescents who took part in longer, 2-h, art-making sessions expressed getting to know themselves better, becoming able to explore their inner selves while noticing their emotions, developing a more positive self-concept, and having a clearer vision of their goals [48]. Self-discovery and increased confidence may be particularly important for this population in helping them develop a clear and optimistic vision of the future.

### Practice implications

Art-based interventions can be beneficial to support youth well-being, as evidenced by the generally positive results found in the present work, and through the previous research findings discussed. For example, participants in this study exhibited how art can be an appropriate and effective means of pushing themselves out of their comfort zone. Indeed, engaging in art making made students realize that they were more capable of coping with difficult emotions than they had previously believed. These findings are consistent with that of previous work amongst youth residing in low income areas, which showed that an expressive art-based intervention improved their confidence [49].

Despite the generally positive findings, the present research also highlights factors that should be considered to promote the acceptability and impact of an art-based intervention for vulnerable youth. Namely, researchers should give careful consideration as a) to what psychological themes are chosen as founding principles within the intervention, and b) what theories or arguments justify the use of said themes. As discussed, the present intervention was co-created alongside the program coordinators; they elaborated on the unique lives and perceived needs of the students. Despite this, neglecting student voices in the creation of the intervention appears to have impacted participation. Regardless of the increase in youth-led movements, which demonstrate their capacity for orchestrating change, existing community-based research does not typically consider youth as full community partners [50, 51]. Youth are typically excluded from the research process because our prevailing views, policies, and practices regarding youth are deficit-focused [52]. However, existing community-based participatory research which provides youth with a leading role in the research process has found that partnering with youth can enhance the success and sustainability of adolescent health interventions [51]. As such, researchers seeking to conduct work with vulnerable youth should meet with them prior to the onset of the intervention to a) gain a sense of the youths’ needs, rather than relying solely on the ideas of the adults close to them, and b) begin developing trust and rapport with them.

In addition to overlooking students’ voices in the research design, some students’ focus on aesthetic quality of their art also provided an important lesson learned. Indeed, attributing pride about one’s creations to perceived artistic skill goes against a fundamental component of art-based methods: the importance of focusing on our subjective experiences in the creative process, rather than the finished piece of art [53–55]. Although criticizing one’s art goes against the aim of art-based interventions, it demonstrates the extent to which students focus on performance. The emphasis on performance was consistent throughout some students, one of whom mentioned that they would not want to continue doing art at school because they were being graded on the finished product. As such, future work should aim to help children detach from performance. A solution for doing so is engaging in philosophical discussions about art and beauty. For example, discussing questions such as “what makes art beautiful? Can art be perceived differently by different people? How so? Does art need to be beautiful to be considered art?” could mitigate the focus on performance.

Overall, our findings suggest that those who enthusiastically engaged in the intervention and were willing to push themselves out of their comfort zone without focusing on the aesthetic quality of their art described experiencing positive implications (e.g., increased confidence and happiness). By comparison, as evidenced through individual interviews, those who were not interested in engaging in the intervention and described seeing no value in participating in it did not report exhibiting the same positive results. Similar to any therapeutic method, art-based interventions, although beneficial for those who actively engage, may not be as impactful for those who see little-to-no value in participating. Consistent engagement throughout an art-based intervention, and more specifically the belief that they can yield positive, worthwhile implications, may be the key to experiencing the benefits described by students in the present work.

### Limitations

A considerable limitation of this work is arguably the insufficient inclusion and implementation of students’ needs. As mentioned, designing the present intervention with only the program coordinators’ details of students’ needs limited the relevance, and arguably the impact, of our work. Indeed, by neglecting to include students’ voices, we unintentionally engaged in adultism, a key barrier to youth participation in research. Adultism is “a lack of pro-child social norms that includes negative attitudes about youth, laws that delegitimize youth, and youth internalizing these negative beliefs” [52] (p. 143). Indeed, what is considered healthy youth development has been defined by adult “experts” rather than by, or in collaboration with, youth themselves [52, 56]. Adultism can be combatted by identifying youth in research in a manner that gives them agency and respects their ability to contribute [52], rather than limiting them to their age or lack of experience [50].

Additionally, lack of time and students’ poor behaviour impacted all facets of data collection, particularly art-making sessions. Approximately ten minutes of every twenty-five minute session was dedicated towards settling the group down so that we could conduct the activity. Generally, art-based interventions with youth are typically no shorter than forty-five minutes [27, 46, 47], further solidifying the challenge posed by limited time in this project. Indeed, as the weeks went on, observational data showed that some students would rush through activities because they feared they would lack sufficient time to complete their creation. This led them to put lesser effort into the activities. Additionally, these students would typically begin their homework after the artistic activities and some felt particularly anxious about having enough time to complete it. Notably, students are guaranteed help with their homework at this after-school program, and students may not have been receiving the same support at home. Overall, consistent with previous research (e.g., [27, 46, 47], we advise that future works should ensure that sessions are at least forty-five minutes. Moreover, if art-based interventions are conducted through a community partnership, as in the present work, we would also advise working closely alongside the program coordinators to ensure that their program’s needs are accommodated both through the topics explored in the designed intervention, and through the intervention scheduling. Specifically, if we were to repeat this work, we would have shortened the number of sessions conducted in order to increase each sessions’ length.

An additional limitation, which had noteworthy implications on our ability to draw concrete conclusions regarding the collected data, was students’ varied, somewhat contradictory feedback. Although it should be noted that the metacognition skills needed to deeply reflect on one’s own thoughts and feelings, and the ability to then verbalize those feelings, are still in development in early adolescence [57]. It is also possible that students may have simply experienced short term benefits from the intervention, rather than long term impacts they could reflect on during the interviews which took place after the intervention ended.

## Supplementary Information

Supplementary Material 1.

## Data Availability

The data that support the findings of this study are available on request from the corresponding author, KS. The data are not publicly available due to their containing information that could compromise the privacy of research participants.
