# Supplementary material for: Promoting the mental health and well-being of vulnerable youth through art: an ethnographic evaluation of an art-based intervention for rural Canadian youth
Source: BMC Psychol. 2025 Mar 3;13:182. doi: 10.1186/s40359-025-02476-8 (PMC11874774; doi:10.1186/s40359-025-02476-8)
Supplement: Supplementary file 1 — Supplementary Material 1. [file 40359_2025_2476_MOESM1_ESM.pdf]

Appendix A. Tables

Table 2. Observation grid used during weekly sessions

**Observation guide – 2023**			
Date:	Activity:	Start time:	Stop time:
**Area of observation**	**Observations**		
What are the students' reactions to the activity?			
What are the students' attitudes towards the activity?			
What are the events that happened during the activity or notable events that influenced? (E.g., interruptions, celebrations, etc.)			
What are the different roles that children adopt during the activity?			
What are the interactions between people? (E.g., nature of interactions, reason they			

occur, emotions expressed)
What are the relationships between the students during the activity?
What are people saying and doing?
What are the non-verbal messages?
What is the general atmosphere in each group?
How is the environment structured? (E.g., physical setting, sitting arrangement)

Table 3. Summary of resulting themes and corresponding student verbatims

Qualitative theme	Illustrative quotes and examples
General appreciation and acceptability of intervention	“I [liked] to do the activities because I don’t like doing homework” (participant 3)<br>“it was boring” (participant 23)
Students’ perceived impact of the intervention	“I feel more comfortable. [MB]: Do you feel more comfortable? Ok. With us or with your friends…? [Student]: Well like with the people here” (participant 1)

	“I managed to discover a little bit of myself [with the drawings we did and the meditation]. [JP]: Because you realized these were things you loved doing? [Student]: yes” (participant 10)
Students’ attitudes towards activities	“[JP]: How did you feel while doing the activities? [Student]: Happy [...] [JP]: Yeah, were you happy? Did it feel good to do activities? Or were you just neutral and a little indifferent? [Student]: yeah.” (participant 15)<br>“[JP]: Was there anything you didn't like doing? Like you said to yourself: “ah I really don’t want to do that”? [Student]: No. [JP]: Did you mind doing it even if you found it difficult? [Student]: No, it's a waste of time. (participant 7)<br>“[JP]: Did you have any favorite activities? Activities that you liked less? [Student]: Dancing. [JP]: Dancing, which was your favorite or least favorite activity? [Student]: Least favorite. [JP]: Didn’t you like that? Did you feel uncomfortable? Were you embarrassed? [Student]: Embarrassed. [JP]: You were embarrassed haha ok. However, you and [another student] still managed to do beautiful choreography. [...] [JP]: Did you have fun anyway? Even if it was a little awkward and you stepped out of your comfort zone? [Student]: yeah yeah. [...] [JP]: And your favorite activity? [Student]: The legos, in fact I drew that, because I had forgotten that we had made the legos. [JP]: Why? [Student]: Because I could speak from a place where I felt safe. [JP]: Yes that’s it. So it was the theme of the activity or the activity itself that you liked? [Student]: the theme.” (participant 23)
Students’ perceptions of created art	“Although it was embarrassing, it also kind of felt good to express it” (participant 25)<br>“Well, the important things are the things we do. [MB]: Okay. [Student]: The memory. [MB]: Okay, yes. So do you like what you're doing right now? Ok. Is there a reason or just because it reminds you of positive moments or...? [Student]: It reminds me of positive moments, when I lost a baseball game, I look at my sheet and I say no problem…” (participant 3)<br>“I think the style you use when you’re drawing really makes the emotion pop out. It shows you which emotion you [inaudible] when you don’t even have to say it… For anger I did cross hatching, because it looks really rough, and so I thought that just represented anger. For fear, I did spikey lines. For sadness I did waves because waves represent water, and

	tears are salty like ocean water. And for happiness I just did a lot of bright colours." (participant 25)
Feelings associated to art-making	"I've already done stuff like this, so it's kind of getting annoying" (participant 37)<br>"Everything is just saying how kids feel and like trying to get them to be calmer. [MB]: To be calmer? [Student]: Yeah, basically just checking on how kids feel every five seconds and doing meditations and stuff like that. [MB]: [...] I wouldn't say the aim was to make you feel calmer. I think the aim of this, at least for Kyra's project, is to see if maybe doing certain activities like this have an improvement in life, your well-being, how you feel… [Student]: Mental health? (participant 37)<br>"[I learned] not to be shy when you have to do the dance" (participant 12)
Challenges impacting art-making	"I didn't understand what to do [...] I had no inspiration in my head" (participant 7)

Note. This chart includes all student verbatims and instances during the research project that exemplify each resulting theme.

Appendix B. Guide for semi-structured interviews

1. Introduction

- Description of the research project
- Description of the process and their participation
- Some clarifications:
    - You don't have to answer questions that don't appeal to you
    - You can take time to think before you answer
    - Tell me if you don't understand a question
    - You can use whatever words you want to represent what you think and not what you think I want to hear; there is no wrong answer, what is important to me is to have your perspective
    - Respect and listen to others, we may not think alike, it is important to talk about it if we have a different vision, we don't laugh at other people's perspectives if they are different from ours
- Consent (ask them if they are okay being interviewed)

2. General appreciation of the intervention

We have done several workshops together in the last few weeks, can you tell me what we did? [reminder of workshops].

- Can you start by telling me about your overall experience with the sessions?
- What were your challenges during the sessions?
- Would you like to continue doing art activities at Phelps Helps?
- What would you change about the way the activities are done?
- Were there any activities that you did not understand or where the instructions were not Clear?
- Did you feel comfortable during the activities?
    - [No] What could have made you feel more comfortable?
        - [Yes] What made you feel comfortable?

3. Students’ attitudes towards the activities and their created art

- To what extent do you feel like the amount of homework you had each week effected your participation in the activities?
- What were your favourite and least favourite activities to do in the last few weeks? Why?
- In what situations in your life do you think art activities can help? How can they help?
- Can you tell me about an art piece that you made that stands out to you?
- Why did you choose to discuss this piece, as opposed to other artwork you made?
    - *If they don’t pick the art piece we wanted to discuss, bring it up now*

- In this piece, I was really struck by…
    - How you drew/created _____

4. Perceived impact of the intervention

- During our sessions, I was struck by when you said ____. Can you expand more on this?
- If you think back to before we started the arts activities and now, what differences do you see in how you feel?
    - The emotions you experience?
    - With those who work at Phelps? with your parents? With your siblings?
- Why do you think there have been these changes after the art activities?
- What was most helpful in the art activities?
- This could be experiencing the relaxing effects of doing art, or the contemplative aspect of art making, for example
- What other changes did you notice?
    - At home?
    - At school?
    - With your friends?

5. Identity

- Has your identity changed as you've grown older?
- What does your identity mean to you?

6. Conclusions

- Do you have anything else to add about the activities?
- Do you have any questions for us?
- [Thank them for their participation]
